# Novel vic-dioximes: synthesis, structure characterization, and antimicrobial activity evaluation

**DOI:** 10.3906/kim-2104-24

**Published:** 2021-08-15

**Authors:** Dumitru URECHE, Ion BULHAC, Alexandru CIOCARLAN, Daniel ROSHCA, Lucian LUPASCU, Paulina BOUROSH

**Affiliations:** 1 Institute of Chemistry, Chisinau, Republic of Moldova; 2 Institute of Applied Physics, Chisinau, Republic of Moldova

**Keywords:** vic-dioxime, spectral analysis, X-ray diffraction, antibacterial activity, antifungal activity

## Abstract

The vic-dioximes are compounds with various industrial uses and scientific applications. Many coordination compounds have been synthesized based on vic-dioximes. This study presents the synthesis and full characterization of two vic-dioximes based on dichloroglyoxime, *p*-aminobenzoic acid, and *p*-aminotoluene. Their structures were proved by IR, ^1^H, ^13^C and ^15^N NMR spectral analysis, and single crystal X-ray diffraction. One of the reported vic-dioximes, *bis*(di-*p*-aminotoluene)glyoxime mono-*p*-aminotoluene trihydrate showed good to moderate antimicrobial activity against both nonpathogenic gram-positive and gram-negative bacteria (*Bacillus subtilis* and *Pseudomonas fluorescens*), phytopathogenic (*Xanthomonas campestris*, *Erwinia amylovora*, *E. carotovora*) and the fungi (*Candida utilis* and *Saccharomyces cerevisiae*) at MIC – 70–150 μg/mL.

## 1. Introduction

vic-Dioximes are widely used as general industrial chemical compounds [1], analytical reagents [2,3], model for biological system [4–7], as well as catalysts in various chemical processes [8,9]. Since the early 1900s vic-dioximes have been used extensively as chelating agents in coordination chemistry [10–13]. Even today vic-dioximes and their complexes constitutes an important class with a versatile reactivity [6,8]. Due to the position of two oximic groups, these compounds can exist as three stereoisomeric forms: anti-(*E*,*E*), amfi-(*E*,*Z*) and *sin*-(*Z*,*Z*), which also influence the modality of metal coordination. The most common is the *N*-*N*-chelation coordination mode favored by the anti-(*E*,*E*) form [6,13,14], but the coordination of these ligands via oxygen atoms from oximic groups is also known [15,16]. vic-Dioximes can form coordination compounds in molecular [17,18], monodeprotonated [19–22] and bis-deprotonated [23] forms. In the coordinated state the intramolecular hydrogen bonds between the oxime anions can be replaced with boron compounds (BF_2_^+^, BF^2+^, B(C_6_H_5_)_2_^+^, B(OH)_2_^+^), thus, encapsulating the respective compounds [24–27]. In the literature, are described the vicinal dioximes containing either aliphatic or aromatic amines [28–34], for which creation the dichloroglyoxime (*DClH_2_*) can be used as a precursor. Also, starting from dichloroglyoxime, a new dioximic ligand was synthesized by condensation with the thiolic derivative - octane-1-thiol, and, based on the obtained dioxime, a Ni-(II) complex was synthesized [35].

The condensation reaction of amines or thiols with dichloroglyoxime leads to the formation of different di-, tetra-, poliamino-derivatives or substituted thioglyoximes [17,36]. Through such kind of reactions, a series of new dioximes have been synthesized [17,37–39]. Both, the oxime and coordination compounds obtained based on their basis show a wide range of pharmacological activities, including antibacterial, antifungal and antidepressant [40–42].

The purposes of this work were the synthesis of new vic-dioxime ligands by the condensation of dichloroglyoxime with *p*-aminobenzoic acid (*paba*) and *p*-aminotoluene (*pat*), their structure elucidation using modern methods of analysis and biological activity assessments against seven strains of nonpathogenic and phytopatogenic bacteria and fungi.

## 2. Materials and methods

### 2.1. Materials

All the reactions were conducted at the room temperature or a moderate heating. All the reagents were purchased from Merck and Aldrich and were used without further purification unless noted otherwise.

### 2.2. Methods

Melting points (m.p.) were measured on a Boetius hot stage and are uncorrected. Infrared spectra (IR) were recorded on a FTIR Spectrum-100 Perkin Elmer spectrometer in Nujol (400–4000 cm^−1^) and using ATR technique (650–4000 cm^−1^). The UV-Vis spectra were recorded in methanol on a Perkin Elmer Lambda 25 spectrometer (400–4000 nm), at a concentration of compounds **1** and **2** (*c* = 0,33·10^−5^ mol/L and *c* = 0,23·10^−5^ mol/L). ^1^H, ^13^C and ^15^N NMR spectra were recorded in DMSO-d6 (99.95 %) on a Bruker Avance DRX 400 (400.13, 100.61 and 40.54 MHz). Chemical shifts (*d*) are reported in ppm and are referenced to the residual nondeuterated solvent peak (2.50 ppm for ^1^H and 39.50 ppm for ^13^C). Coupling constants (*J*) are reported in Hertz (Hz). The following abbreviations were used to explain the multiplicities: s = singlet, d = doublet, t = triplet, q = quartet, qvin. = quintet, sex = sextet, m = multiplet, brs = broad singlet. X-ray analyses on single crystal were performed on a Xcalibur E diffractometers with a CCD detector using graphite-monochromatized MoK*_α_* radiation at room temperature.

### 2.3. Synthesis

#### 2.3.1. Synthesis of *bis*(*p*-aminobenzoic acid)-glyoxime hydrate [H_4_L^1^]·H_2_O (1)

The yellow solution resulted after dissolving of dichloroglyoxime (0.31 g, 0.02 mol) and *p*-aminobenzoic acid (0.55 g, 0.04 mol) in MeOH (10 mL) was stirred for 15 min. Then, to the reaction mixture, consecutively, Na_2_CO_3_ (0.21 g, 0.02 mol) was added and after 15 min H_2_O (3 mL) was added with additional stirring for 2 h. As result, a radish-yellow sediment was obtained, then filtered through a glass filter and washed, consecutively, with MeOH and Et_2_O. After drying, a beige product (0.395 g, 56%) soluble in DMF and DMSO was obtained. The filtrate has been passed into a chemical beaker and allowed to crystallize at room temperature. After six days, yellowish needle shaped crystals were obtained. m.p. 281–284 °C. Anal. Calcd. for C_16_H_16_N_4_O_7_ (376.23): (430.09): C, 51.07; H, 4.28; N, 14.89. Found: C, 50.93; H, 4.30; N, 14.79. IR (n_max_/cm^−1^): 3535 (ν(OH)_H2O_), 3180 (ν(OH)_oxime_+ ν(NH)), 1677 (ν(C=O)), 1652 (ν(C=N)), 1600, 1519, 1459 (ν(C=C)), 854 (δ(CH)_arom. nonplan._). ^1^HNMR (400.13 MHz, DMSO-d6, ppm): d 10.92 (l.s., 2H, CO_2_H), 8.75 (s, 2H, NH), 7.66 (d, J=8.8 Hz, 4H, C_2_-C_6_, C_2_′-C_6_′), 6.82 (d, J=8.8 Hz, 4H, C_3_-C_5_, C_3_′-C_5_′). _13_CNMR (100.61 MHz, DMSO-d6, ppm) d 167.6 (COOH), 144.55 (C_4_, C_4_′), 142.32 (C_8_, C_9_), 130.45 (C_2_-C_6_, C_2_′-C_6_′), 123.19 (C_1_, C_1_′), 117.92 (C_3_-C_5_, C_3_′-C_5_′). ^15^NNMR (40.54 MHz, DMSO-d6, ppm): d 100.9.

#### 2.3.2. Synthesis of *bis*(di-*p*-aminotoluene)-glyoxime mono-p-aminotoluene trihydrate [(H_2_L^2^)_2_] pat·3H_2_O (2)

The yellow solution resulted after dissolving of dichloroglyoxime (0.31 g, 0,02 mol) and *p*-aminotoluene (0.48 g, 0,04 mol) in EtOH (10 mL) was stirred for 15 min. Then, to the reaction mixture, consecutively, Na_2_CO_3_ (0.21 g, 0.02 mol) was added and after 15 min H_2_O (3 mL) was added. The reaction mixture was heated at 50 °C for 40 min until complete carbonate dissolving, then heating was stopped and it was stirred for 1.5 h. As result, a white coloured sediment was obtained, then filtered through a glass filter and washed with Et_2_O. After drying, it was obtained a beige product (0.78 g, 53%) soluble in DMF, MeOH, EtOH, DMSO and insoluble in H_2_O. The filtrate has been passed into a chemical beaker and allowed to crystallize at room temperature. After one week, radish needle shaped crystals were obtained. m.p. 205–207 °C. Anal. Calcd. for C_39_H_51_N_9_O_7_ (757.89):C, 61.80; H, 6.78; N, 16.64. Found: C, 61.93; H, 6.71; N, 16.72. IR (n_max_/cm^−1^): 3676 (ν(OH) _oxime_), 3371 (ν_as_(NH_2_)), 3308 (ν_s_(NH_2_)), 3187 (ν(NH)), 1614 (δ(NH_2_)), 1516, 1452, 1407 (ν(C=C)), 812 (δ(CH)_arom.nonplan._). ^1^HNMR (400.13 MHz, DMSO-d6, ppm) d 10.36 (l.s., 2H, >N-OH), 8.44 (s, 2H, NH), 6.88 (d, J=8.4 Hz, 4H, C_3_-C_5_, C_3_′-C_5_′), 6.67 (d, J=8.4 Hz, 4H, C_2_-C_6_, C_2_′-C_6_′), 2.16 (s, 6H, 2xC_7_,_7_′-H_3_). ^13^CNMR (100.61 MHz, DMSO-d6, ppm) d 143.56 (C_1_, C_1_′), 137.43 (C_8_, C_9_), 131.05 (C_4_, C_4_′), 129.26 (C_3_-C_5_, C_3_′-C_5_′), 119.77 (C_2_-C_6_, C_2_′-C_6_′), 20.64 (C_7_, C_7_′). ^15^NNMR (40.54 MHz, DMSO-d6, ppm): d 97.1.

### 2.4. Microbiological activity assessments

Antimicrobial activity evaluation of both compounds was performed on the following microorganisms: nonpathogenic gram-positive and gram-negative strains of *Bacillus subtilis* NCNM BB-01 (ATCC 33608) and *Pseudomonas fluorescens* NCNM PFB-01 (ATCC 25323), respectively, and phytopathogenic strains of *Xanthomonas campestris* NCNM BX-01 (ATCC 53196), *Erwinia amylovora* NCNM BE-01 (ATCC 29780), *E. carotovora* NCNM BE-03 (ATCC 15713), as well on the fungi strains of *Candida utilis* NCNM Y-22 (ATCC 44638) and *Saccharomyces cerevisiae* NCNM Y-20 (ATCC 4117).

Before the evaluation of the antimicrobial activity of the compounds **1** and **2** the microbial cell viability assessment was done on the used microorganisms. Moreover, this assessment is performed periodically and mandatory in the process of maintaining the microorganisms in the collection.

For testing the double successive dilution method was used as reported before [43]. For this, at the initial stage, 1 mL of peptone broth for test bacteria and Sabouraud broth for test fungi was introduced into a series of 10 tubes. Subsequently, 1 mL of the analyzed preparation was dropped into the first test tube. Then, the obtained mixture was pipetted, and 1 mL of it was transferred to the next tube, so the procedure was repeated until tube no. 10 of the series. Thus, the concentration of the initial preparation decreased 2-fold in each subsequent tube. At the same time, 24 h test microorganisms cultures were prepared. Initially, suspensions of test microorganisms were prepared with optical densities (O.D.) of 2.0 for tested bacteria and 7.0 for fungi according to the McFarland index. Subsequently, 1 mL of the obtained microbial suspension was dropped in a tube containing 9 mL of sterile distilled water. The content of the tube was mixed, and 1 mL of the mixture was transferred to tube no. 2 of the 5-tube series containing 9 mL of sterile distilled water. From the 5-th tubes of the series, 0.1 mL of the microbial suspension was taken, which represent the seeded dose and added to each tube with titrated preparation. Subsequently, the tubes with titrated preparation and the seeded doses of the microorganisms were kept in the thermostat at 35 °C for 24 h. On the second day, a preliminary analysis of the results was made. The last tube from the series in which no visible growth of microorganisms has been detected is considered to be the minimal inhibitory concentration (MIC) of the preparation. For the estimation of the minimal bactericidal and fungicidal concentrations, the contents of the test tubes with MIC and with higher concentrations are seeded on peptone and Sabouraud agar from Petri dishes with the use of the bacteriological loop. The seeded dishes are kept in the thermostat at 35 °C for 24 h. The concentration of preparation, which does not allow the growth of any colony of microorganisms, is considered to be the minimal bactericidal and fungicidal concentrations of the preparation.

### 2.5. Crystallographic studies

Determination of the unit cell parameters and processing of experimental data were performed using the CrysAlis Oxford Diffraction Ltd. (CrysAlis RED, O.D.L. Version 1.171.34.76. 2003). The structures were solved by direct methods and refined by full-matrix least-squares on weighted *F*^2^ values for all reflections using the *SHELXL2014* suite of programs [44]. All non-H atoms in the compounds were refined with anisotropic displacement parameters. The positions of hydrogen atoms in the structures were located on difference Fourier maps or calculated geometrically and refined isotropically in the “rigid body” model with *U* = 1.2*U*_eq_ or 1.5*U*_eq_ of corresponding O, N, and C atoms. Crystallographic data and structure refinements are summarized in Table S1, and the details of the hydrogen bonding interactions are given in Table S2. CCDC 2051228 and 2051229 contain the supplementary crystallographic data for this paper. These data can be obtained free of charge via www.ccdc.cam.ac.uk/data_request/cif, or by emailing data_request@ccdc.cam.ac.uk, or by contacting The Cambridge Crystallographic Data Centre, 12, Union Road, Cambridge CB2 1EZ, UK; fax: +44 1223 336033.

## 3. Results and discussion

The syntheses of target compounds were performed by interaction of dichloroglyoxime (DClH_2_) with *p*-aminobenzoic acid (*paba*) and *p*-aminotoluene (*pat*), in 1:2 molar ratio, in methanol and ethanol, respectively, according to Scheme. The new vic-dioxime bis(*p*-aminobenzoic acid)-glyoxime hydrate (H_4_L^1^·H_2_O, **1**) and bis(di-*p*-aminotoluene)-glyoxime mono-*p*-aminotoluene trihydrate ((H_2_L^2^)_2_·pat·3H_2_O, **2**) were obtained in 56% and 53% yields, and their structures were confirmed by spectral IR, ^1^H, ^13^C and ^15^N NMR analyses and single crystal X-ray diffraction.

### 3.1. FT IR and UV-Vis spectroscopy

The region 3800–2400 cm^−1^ from the IR spectrum of compound **1** is the most informative and shows multiple strong absorption bands. The absorption maximum from 3535 cm^−1^ may be attributed to the ν(OH)_H2O_ vibration with the formation of hydrogen bonds [45], and narrow band from 3361 cm^−1^ as the first overtone of the ν(C=O) group vibration [46]. The presence of oximic OH group is confirmed by a large peak at 3180 cm^−1^, which is typical also for ν(NH), the value and the width of which proves the association of both mentioned groups by hydrogen bonds [45,47]. Also, the signal from 1626 cm^−1^ belongs to the δ(NH) vibrations of amino group.

A series of middle intensity absorption bands (in the range 3100–2700 cm^−1^) may be attributed to the ν(CH) vibrations of the aromatic rings, and their intensity higher than usual is caused by the relatively large number of aromatic rings in the complex [45]. The ν(C=C) vibrations at 1600, 1519 and 1459 cm^−1^, but also δ(CH)_nonpl._ oscillation from 854 cm^−1^ represents 1,4-substituted aromatic rings (two neighboring hydrogen atoms) [45,48].

The spectrum of compounds **2** has many similarities with that of compound **1**, but it has also some differences related to the absence of the carboxylic group and presence of –CH_3_ group from oxime and –NH_2_ group from molecule of *p*-aminotoluene, which co-crystallizes with oxime molecule. The main vibrations characteristic for amino group ν_as_(NH_2_) and ν_s_(NH_2_) are visible at 3371 and 3308 cm^−1^, respectively, and that of δ(NH_2_) is present at 1614 cm^−1^. The signals of the aliphatic groups (–CH_3_) are located in the range of 3000–2700 cm^−1^, as well as those for δ_as_(CH_3_) and δ_s_(CH_3_) at 1466 and 1380 cm^−1^, respectively. The IR spectrum of compound **2** contains some strong absorption bands specific for dimeric carboxylic acids whose –OH groups form hydrogen bonds of O-H…O type at 2672 and 2531 cm^−1^ [43]. The peak of ν(C=O) groups is well seen at 1677 cm^−1^, this of ν(C-O)+δ(OH)_plan._ groups at 1421 cm^−1^ and δ(OH)_nonpl._ from dimer at 898 cm^−1^. The signal of ν(C=N)_oxime_ is visible at 1652 cm^−1^ [44]. The vibration from 812 cm^−1^ proves the presence of 1,4-substituted aromatic rings (two neighboring hydrogen atoms) [45].

**Figure 1:**
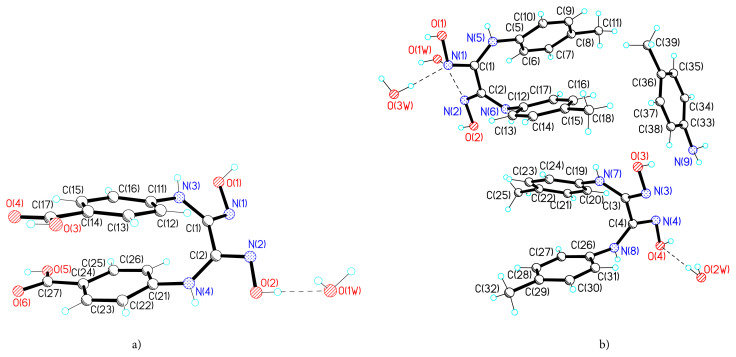
Molecular structure of compounds **1** (a) and **2** (b).

In the UV-Vis spectra of dioximes **1** and **2**, acquired in methanol, two pairs of absorption maximums are observed at 225 and 230 nm, 295 and 340 nm, respectively. First peaks can be assigned to π→π* type transactions in benzene ring (C=C bonds) and to azomethine groups (-C=N) from the oximic unit (Figure 1) [49]. The second pair of peaks represent n→π* type transactions characteristic for iminic groups (-C=N:-) and carbonyl groups (-C=O:) [49, 50].

### 3.2. NMR characterization

The NMR analysis fully confirmed the structure of compounds **1** and **2**. Thus, the doublets from 6.82 and 7.66 ppm belong to unsubstituted protons from aromatic rings of ligand **1**. The protons from NH groups appeared at 8.75 ppm and those from oximic groups at 10.92 ppm, all as large singlets. The tertiary carbon atoms from aromatic rings were registered at 117.92 and 130.45 ppm, quaternary at 123.19 and 144.55 ppm. The signal of carbons from carboxylic and oximic groups is localized at 142.32 and 167.60 ppm.

According to NMR data, the unsubstituted aromatic protons from the molecule of compound **2** are visible at 6.67 and 6.88 ppm. The protons of methyl groups appear at 2.15 ppm and those from NH groups at 8.43 ppm, all as singlets. The protons from oximic groups were registered as large singlet at 10.36 ppm. The tertiary carbons from aromatic rings were registered at 119.77 and 129.26 ppm, while quaternary at 131.05 and 143.56 ppm. The signals from 137.43 ppm belong s to oximic carbon atoms.

The ^15^N NMR spectra of compounds **1** and **2** contain the signals of aminic N atoms at 100.9 ppm and 97.1 ppm, respectively.

### 3.3. Single crystal structures

The recent literature review in CSD [51] revealed that only two polymorphs of dianylineglyoxime salts as uncoordinated dioxime with “aromatic wings” are known [17]. We reported new di-aminobenzoylglioxime compounds **1** and **2** with substituents –COOH and –CH_3_ in *para*-positions crystallized in centrosymmetric *C*2/*c* (**1**) and uncentered *Pn* (**2**) monoclinic space groups (Table S1).

In the asymmetrical part of the unit cell of the crystal **1** a molecule of H_4_L^1^ and water were detected, and in that of crystal **2** - two molecules of H_2_L^2^, one crystallization molecule of 4-methylaniline (*p*-toluidine) L^3^ and three molecules of water, as a result their formulas, can be described as H_4_L^1^·H_2_O and (H_2_L^2^)_2_·L^3^·3H_2_O (Figures 1a and 1b). It should be mentioned, that the title compounds were never reported before. A careful search in CSD showed a Fe(II) complex with ligand, which contains two fragments of methylated dianylineglyoxime being united by three BF^2+^ moieties [25,52]. In CSD there are nine cases when *p*-aminotoluene crystallizes with diverse molecules, inclusive derivatives of 4-nitrophthalic acid or of 3,3′-(pentane-1,5-diylbis(oxy))-bis-(5-methoxybenzoic) acid with *pat* [53, 54].

**Figure 2:**
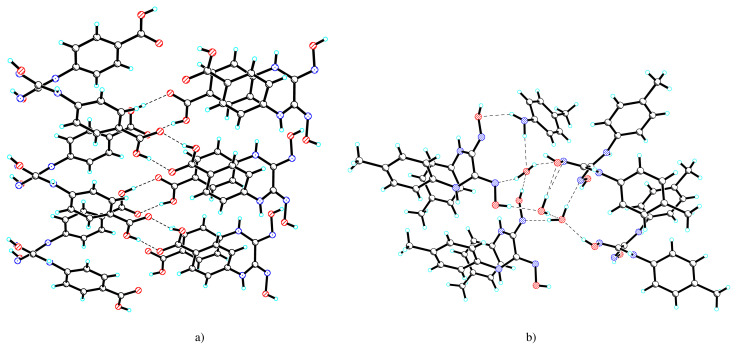
Fragment of chains in **1** (a) and view of H-bonded components in **2** (b).

**Figure 3:**
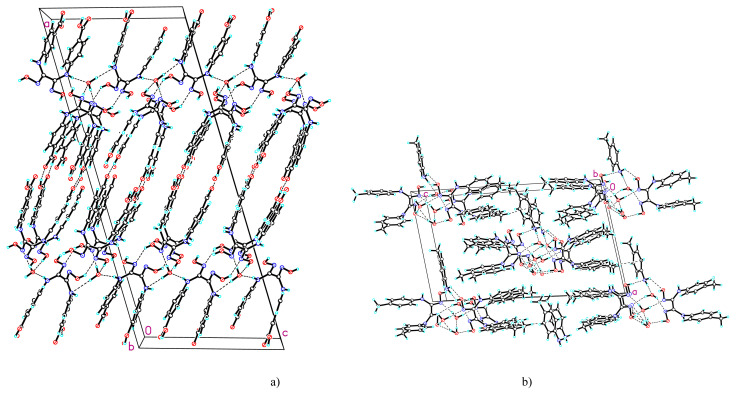
Fragments of crystal packing in **1** (a) and **2** (b) along *b*.

Due the fact that these neutral molecules possess both proton donor groups and acceptor atoms, in respective crystals, the components are connected by complicated system of hydrogen bonds (Table S2). In the crystal of compound **1** can be highlighted chains parallel with plane *b*, formed by synthons R(8)^2^_2_ via –COOH groups of neighboring H_2_L (Figure 2a), which further develops into a 3D network via oximic groups and water molecules (Figure 3a).

In the crystal of compound **2** three water molecules unite four molecules of H_2_L_2_ and one of *pat* (Figure 2b), which further develops in parallel chains with *a*, united via fine C-H...X bonds, where X is the center of the aromatic ring from molecule *pat* (C...X 3.625, H...X 3.087 Å) (Figure 3b).

### 3.4. Antimicrobial activity

A detailed literature search in the field of biological activity of vic-dioximes offered a few related bibliographic references. In one of them, the authors reported that, in contrast to its metal Cu^II^, Ni^II^, Zn^II^, Cd^II^ complexes, vic-dixime ligand bearing one naphthyl disodium disulfonate unite has no inhibitory effects on the growth of *Rhodotorula rubra*, *Kluyveromyces marxianus*, *Aspergillus fumigatus* and *Mucor pusillus* fungi strains [41].

Another research group performed an in vitro assessments of some 3- and 4-substituted benzaldehyde hydrazones vic-ligands and their Cu^II^, Ni^II^, Co^II^ complexes on 18 strains of bacteria and yeasts [55]. The authors mentioned that all the tested compounds exhibit moderate antimicrobial activities, the ligands being generally more active. Here must be mentioned mono-3-methylbenzaldehyde hydrazone vic-dioxime, followed by mono-4-methoxybenzaldehyde hydrazone vic-dioxime. These ligands have shown slightly higher activities against *Bacillus thrungiensis* and strong activity against *Candida utilis*, *C. albicans*, *C. glabata*, *C. trophicalis*, S*accharomyces cerevisiae* compared to the reference compounds.

A comparative study of the antibacterial activity of two vic-dioxime ligands containing one *p*-tolyl and benzyl-piperazinyl, and bis-benzyl-piperazinyl radicals and their Ni^II^, Cu^II^, Co^II^, Zn^II^ metal complexes was performed by authors [56]. According to them only ligand bearing bis-benzyl-piperazinyl units have shown weak antibacterial activity.

The in vitro growth inhibitory activity of the vic-dioxime [H_4_L^1^]·H_2_O **1** and [H_2_L^2^]·*pat*·3H_2_O **2** ligands was assessed against both nonpathogenic gram-positive and gram-negative bacteria (*Bacillus subtilis* and *Pseudomonas fluorescens*), phytopathogenic (*Xanthomonas campestris*, *Erwinia amylovora*, *E. carotovora*) and the fungi (*Candida utilis* and *Saccharomyces cerevisiae*) (Table).

Compound **2** exhibits average antibacterial and antifungal properties in the range of concentrations of 70–150 μg/mL for bacteria and fungi. In contrast to other vic-dioxime ligands that do not show antibacterial and antifungal activity compared to their metal complexes [41, 57], we managed to obtain reported ligands in crystalline form and in higher yields using simple but efficient methods of synthesis. Looking to the data presented in Table, it is well seen that compound **2** exhibits variable biological activity depending on the bacterial or fungicidal species. A possible cause of this variation could be the different permeability of the cells of the microorganism or the difference between the ribosomes of the microbial cells [58–61]. In order to avoid speculation about the influence of the co-crystallized *p*-aminotoluene molecule on the activity of compound **2**, a separate evaluation was performed on said bacterial and fungal strains. According to this, *p*-aminotoluene did not show antimicrobial activity.

A probable explanation of the different activity of compound **1** and **2** may be the presence of different substituents in the benzene ring, otherwise their structure being identical. The inactivity of compound **1** may be due to the presence of the carboxyl group in the para-position of the benzene ring, make this compound highly hydrophilic and, consequently, decrease the cell membrane penetration. In the case of compound **2**, the situation is different; the presence of the methyl group in the para-position of the benzene ring increase the lipophilic nature of this compound, and consequently increase its cell membrane penetration capacity.

## 4. Conclusion

As result of this research *bis*(*p*-aminobenzoic acid)-glyoxime hydrate and *bis*(di-*p*-aminotoluene)glyoxime mono-*p*-aminotoluene trihydrate were synthetized and fully characterized, including by single crystal X-ray diffraction. The last reported compound showed good antimicrobial activity against five species of gram-positive, gram-negative, phytopatogenic bacteria, and two strains of fungi. The reported data are a good contribution to the chemistry of vic-dioximes, and the compounds obtained are promising ligands for the synthesis of metal complexes.

**Table S1:** Crystal data and structure refinement for compounds **1** and **2**.

	1	2
Formula	C _16_H _16_N _4_O _7_	C _39_H _51_N _9_O _7_
M _r_	376.33	757.89
Cryst. system	monoclinic	monoclinic
Space group	*C*2/ *c*	*Pn*
*а* / Å	35.338(2)	13.863(2)
*b* / Å	6.8836(4)	6.4122(14)
*с* / Å	14.3090(9)	23.213(5)
*b* / °	106.419(7)	99.512(18)
*V*, Å ^3^	3338.7(4)	2035.1(7)
*Z*	8	2
*D* _calcd_ / g/c	1.497	1.237
*μ* / mm ^−^ ^1^	0.120	0.087
*F*(000)	1568	808
Crystal size / mm ^3^	0.40 x 0.08 x 0.04	0.50 x 0.06 x 0.04
Reflections collected / independent reflections	5258/2938 ( *R* _int_ = 0.0312)	7811/5837 ( *R* _int_ = 0.0682)
Completeness to theta / % ( *θ* = 25.05)	99.3	99.8
Parameters	244	510
Goodness-of-fit on *F* ^2^	1.004	1.007
final *R* _1_, *wR* _2_	*R* _1_ = 0.0522, *wR* _2_ = 0.1174	*R* _1_ = 0.0737, *wR* _2_ = 0.1349
*R* indices (all data)	*R* _1_ = = 0.0890, *wR* _2_ = 0.1315	*R* _1_ = 0.2073, *wR* _2_ = 0.1988
Largest diff. peak and hole, *e*×Å ^−^ ^3^	0.263, −0.292	0.233, −0.214

**Table S2:** Hydrogen bond distances (Å) and angles (°) in molecules **1** and **2**.

D–H···A	*d*(H···A)	*d*(D···A)	Đ(DHA)	Symmetry transformations for A
**1**				
O(1)–H(1)⋯O(2)	2.65	3.449(3)	166	− *x*+1/2, *y*+1/2, − *z*+3/2
O(1)–H(1)⋯N(2)	2.03	2.746(3)	145	− *x*+1/2, *y*+1/2, − *z*+3/2
O(2)–H(2)⋯O(1W)	1.99	2.809(2)	175	*x*, *y*, *z*
O(3)–H(3)⋯O(6)	1.82	2.624(2)	165	− *x*, *y*−1, − *z*+1/2
O(5)–H(5)⋯O(4)	1.78	2.593(2)	169	− *x*, *y*+1, − *z*+1/2
N(4)–H(4)⋯O(1W)	2.18	3.031(3)	169	− *x*+1/2, − *y*+1/2, − *z*+1
O(1W)–H(1)⋯N(1)	1.93	2.824(3)	171	*x*, *y*−1, *z*
O(1W)–H(2)⋯N(3)	2.42	3.405(3)	170	− *x*+1/2, *y*−1/2, − *z*+3/2
**2**				
N(9)–H(9A)⋯O(1W)	2.19	3.14(1)	178	*x*+1/2, − *y*, *z*−1/2
N(9)–H(9B)⋯O(1)	2.52	3.18(2)	132	*x*+1/2, − *y*, *z*−1/2
O(1)–H(1)⋯O(1W)	1.83	2.64(1)	172	*x*, *y*+1, *z*
O(2)–H(2)⋯O(2W)	1.90	3.70(1)	176	*x*−1/2, − *y*, *z*+1/2
O(3)–H(3)⋯N(9)	1.93	2.72(1)	162	*x*, *y*+1, *z*
O(4)–H(4)⋯O(3W)	1.83	3.64(1)	170	*x*+1/2, − *y*, *z*−1/2
O(1W)–H(1)⋯N(2)	2.09	2.82(1)	149	*x*, *y*, *z*
O(1W)–H(2)⋯N(4)	2.10	2.86(1)	156	*x*+1/2, − *y*+1, *z*−1/2
O(2W)–H(1)⋯O(2W)	2.00	2.88(1)	178	*x*, *y*, *z*
O(2W)–H(2)⋯O(4)	2.09	2.97(2)	179	*x*, *y*, *z*
O(2W)–H(2)⋯N(4)	2.60	3.37(1)	147	*x*, *y*, *z*
O(3W)–H(1)⋯N(1)	1.96	2.83(1)	177	*x*, *y*, *z*
O(3W)–H(2)⋯N(3)	1.94	3.82(1)	160	*x*−1/2, − *y*+1, *z*+1/2

## Figures and Tables

**Scheme:**
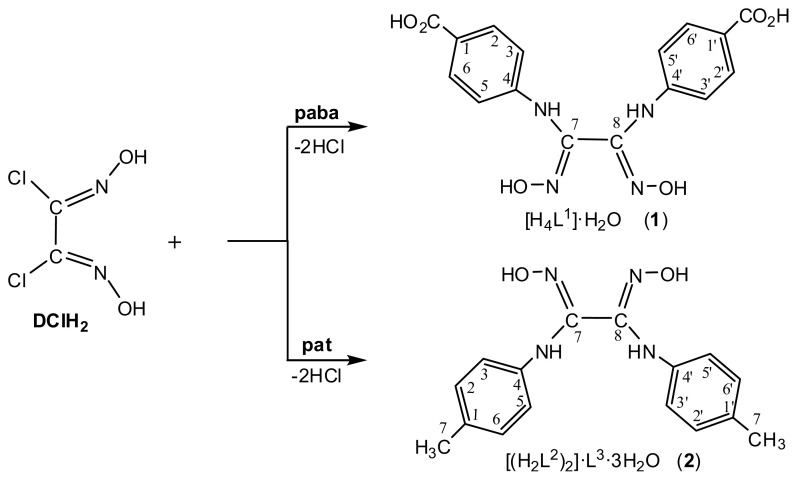
Synthesis of vic-dioximes **1** and **2**.

**Table:** In vitro antifungal and antibacterial activities of compound **1** and **2**.

	MBC and MFC, mg/mL						
Compd	*Bacillus subtilis*	*Pseudomonas fluorescens*	*Erwinia amylovora*	*Erwinia carotovora*	*Xanthomonas campestris*	*Candida Utilis*	*Saccharomuces cerevisiae*
**1**	N/A	N/A	N/A	N/A	N/A	N/A	N/A
**2**	70	150	70	150	150	70	150

MBC – *minimal bactericidal concentration*;

MFC - *minimal fungicidal concentration;*

N/A – non active
